# Pharmacological Treatment of Type-2-Diabetes and Cardiovascular Comorbidities: Differences between Undocumented Migrants and Natives in Italy

**DOI:** 10.3390/healthcare11010004

**Published:** 2022-12-20

**Authors:** Gianfrancesco Fiorini, Giacomo Pellegrini, Matteo Franchi, Angela Ida Pincelli, Antonello Emilio Rigamonti, Giovanni Corrao, Alessandro Sartorio, Silvano Gabriele Cella

**Affiliations:** 1 Istituti Clinici Zucchi, Gruppo San Donato, 20900 Monza, Italy; 2 National Centre for Healthcare Research and Pharmacoepidemiology, 20126 Milan, Italy; 3 Laboratory of Healthcare Research and Pharmacoepidemiology, Department of Statistics and Quantitative Methods, University of Milano Bicocca, 20126 Milan, Italy; 4 Endocrinology and Diabetes Center, San Gerardo Hospital, 20900 Monza, Italy; 5 Laboratory of Clinical Pharmacology and Pharmacoepidemiology, Department of Clinical Sciences and Community Health, University of Milan, 20122 Milan, Italy; 6 Istituto Auxologico Italiano, IRCCS, Experimental Laboratory for Auxo-Endocrinological Research, 28824 Piancavallo-Verbania, Italy

**Keywords:** diabetes, undocumented migrants, cardiovascular diseases, antidiabetic agents

## Abstract

Diabetes prevalence is growing worldwide, especially in some populations. Though migrations seem to contribute to the presence in host countries of a significant number of patients with diabetes and its comorbidities, very little is known about the health conditions of undocumented migrants. We retrospectively studied 838 patients with type 2 diabetes mellitus (T2DM), 425 Italians followed by the diabetes clinic of a university hospital, and 413 undocumented migrants receiving assistance from a non-governmental organization. We analyzed their demographic and clinical data together with the medications they were on. The prevalence of the use of specific classes of drugs was compared between undocumented migrants and Italians by fitting a Poisson regression model, and the results were reported as prevalence rate ratios (PRRs) with a 95% confidence interval. Undocumented migrants with T2DM received fewer medications for cardiovascular (CV) conditions (PRR: 0.68, 0.60 to 0.76) than Italians, after correcting for confounding factors. Only sulfonylureas were more frequently used in undocumented migrants. The causes of these differences are not completely clear, but social, cultural, and economic factors can have an important role. More efforts are needed to provide appropriate treatment of diabetes and its CV comorbidities to undocumented migrants.

## 1. Introduction

The prevalence of T2DM continues to rise all over the world, especially in low-income and developing countries [1]. This increase is associated with an increasing prevalence of obesity. Both are mainly due to a low energy expenditure associated with excessive caloric intake. Diabetic patients frequently have a number of comorbidities, among which CV diseases have a considerable weight. They affect at least one third of persons with T2DM [2] and are responsible for death in three quarters of diabetics aged over 40; moreover, diabetics are more likely than non-diabetics to die from their first cardiovascular event [3].

The prevalence of arterial hypertension is also continually growing; this condition frequently coexists with T2DM. This association is not casual, since both conditions share some pathophysiologic aspects, especially those related to insulin resistance and obesity [4]. While hypertension occurs in 50 to 80% of persons with T2DM, T2DM is almost 2.5 times more frequent in hypertensive patients than in those with normal blood pressure; hypertension is a risk factor for diabetes and often precedes its onset [5]. The association among T2DM, hypertension, and CV diseases has been recognized for many years. For example, the Framingham Heart Study had already demonstrated that T2DM increases the risk of peripheral arterial disease, hypertension, and myocardial infarction from two to four times [6]. Hypertension has long been known to be an important risk factor for microvascular damage, renal disease, CV diseases, and stroke [7]. All of these are also known to be potential complications of T2DM.

In addition, gender and ethnicity have a role in the incidence of diabetes and hypertension. Hypertension is more common in Black than in White populations between the ages of 45 and 74 years [8]. Black persons are also known to have a 77% higher prevalence of diabetes than White persons in the United States, while among Hispanic adults, the prevalence is 66% higher than that of the White population [9]. However, the contribution of psychosocial, socioeconomic, and local environmental factors to these differences has to be considered [9].

In recent years, many novel pharmacological strategies, targeting the pathogenetic mechanisms of both T2DM and CV diseases, have been made available, but according to the recommendations of ADA 2022 clinical practice guidelines, the first step in preventing the progression of hypertension and diabetes, mainly in patients with both conditions, remains non-pharmacological interventions, such as limitation of caloric intake, weight loss, and physical activity [10]. Unfortunately, these interventions are often difficult to implement, though they have beneficial effects not only for patients but also in terms of savings in public health costs [11].

Given the high prevalence of T2DM, its many complications, its entanglement with a number of different chronic conditions, and the high costs posed to public health expenditure, it deserves greater attention. This is especially true in our multi-ethnic Western countries, where, in the last few decades, many immigrants have arrived from extremely disadvantaged areas. If they are undocumented, they are unable to access all of the health and prevention assessment and intervention measures. While the results of many studies on documented migrants are now available, very few data are available on the health conditions of undocumented migrants. However, it is known that they have a significant burden of chronic conditions [12], including diabetes and its comorbidities [13].

In Italy, diabetic undocumented migrants can receive free medical assistance through charitable health institutions. However, due to different factors, including the many problems that undocumented migrants have to face, it cannot be ruled out that they receive inadequate pharmacologic therapy. With the present study, we evaluated if there are differences in the treatment of T2DM and its complications in a sample of undocumented migrants assisted by a major charity and in a comparable group of Italian diabetic patients attending a clinic of the Italian National Health Service.

## 2. Materials and Methods

### 2.1. Patients

We compared two groups of patients with T2DM, one of undocumented migrants and one of Italian patients. The first (*N* = 413) was composed of all the patients with diabetes who had attended the outpatient clinic of Opera San Francesco (OSF) during 2018. This is the most important charity giving medical assistance for free to undocumented migrants in Lombardy, Italy. It runs both general medicine and specialized clinics, where volunteer doctors see patients and prescribe them the necessary tests and medications. The latter are dispensed directly to the patient by the internal pharmacy. These medications are in part obtained for free from donors and in part purchased by OSF with money coming from donations. The same medications available for Italian patients were also available for those of OSF. Complete electronic records of all this activity are available dating back to 2011. The second group was made of 425 Italian patients randomly chosen among those attending the diabetes clinic of a university hospital in Lombardy, Italy (Ospedale San Gerardo).

No selection or exclusion criteria were applied. Demographic, clinical, and laboratory data were collected, together with information on personal history and lifestyles; the Q-score, an indicator of good quality of care ranging between 0 and 40 (maximum control of diabetes), was calculated as suggested [14]. 

### 2.2. Medicine

The medications dispensed to the patients of Opera San Francesco and those prescribed to the patients of Ospedale San Gerardo were grouped on the basis of the anatomical, therapeutic, chemical (ATC) classification, as previously described [12]. All the medications for each patient were recorded, then we separately analyzed those used to treat diabetes and those used for comorbidities. Among the latter, CV drugs were considered separately. 

### 2.3. Statistical Analysis

Baseline characteristics were reported using frequencies and percentages for categorical variables and using the median (with interquartile range) or mean (with standard deviation) for continuous variables. Differences between Italians and undocumented migrants were calculated for categorical variables by the Chi-square test or the Fisher test, as appropriate, and for continuous variables by the Wilcoxon test or the t-test, as appropriate. The *p*-values were calculated among subjects with non-missing information.

The prevalence of use of specific classes of drugs was compared between undocumented migrants and Italians, by fitting a Poisson regression model, and the results were reported as prevalence rate ratio (PRR) with a 95% confidence interval.

In order to reduce confounding factors, the models were also adjusted for age, sex, the presence of cardiovascular disease risk factors, the presence of a cardiovascular disease, nephropathy, retinopathy, ulcers, and the Q-score (considered as a five-unit increase).

In order to evaluate the impact of missing data on the estimates obtained in the main analysis, a sensitivity analysis was conducted calculating PRRs excluding the variables with the higher rate of missing data, i.e., nephropathy, retinopathy, and ulcers. 

## 3. Results

The two groups of patients had many differences, not only in the phenotype and clinical features of diabetes, but also in socio-demographic characteristics (Table 1). 

### 3.1. Antidiabetic Drugs

Table 2 shows antidiabetic medications used in the two groups of patients. 

As can be seen, the percentage of patients receiving oral antidiabetic agents was higher in Italians. This was mainly evident for metformin and sodium–glucose cotransporter 2 (SGLT2) inhibitors, while sulfonylureas were more frequently prescribed to migrants. Dipeptidyl peptidase 4 (DPP-4) inhibitors were prescribed with the same frequency in both groups. For injective therapy, intermediate- and long-acting insulins, and glucagon-like peptide 1 (GLP-1) analogues were more frequently prescribed to Italians.

**Figure 1:**
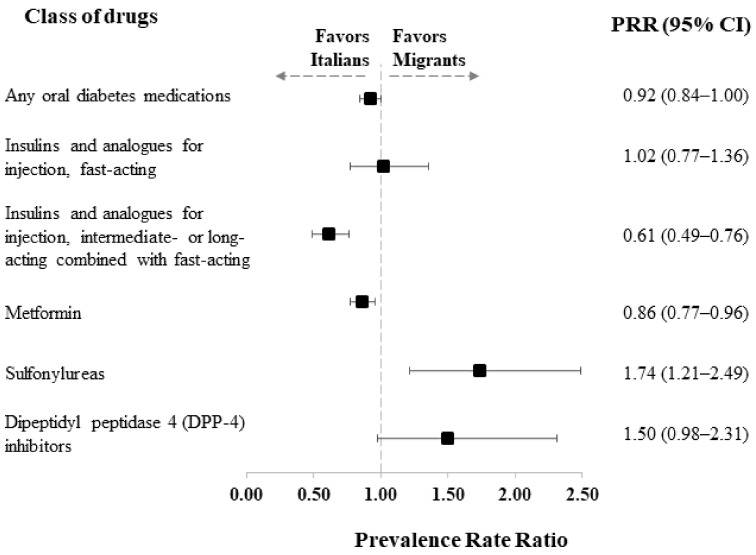
Prevalence rate ratio (PRR), along with 95% confidence intervals, estimated from a Poisson regression model for comparing the prevalence of use of selected antidiabetic drugs between undocumented migrants and Italians, adjusted for patients’ characteristics (age class, sex, comorbidities, and Q-score).

Figure 1 shows the PRRs of the use of the different classes of antidiabetic drugs in migrants compared to Italians. 

The PRRs could not be carried out for all the different drugs due to the small numerosity of some classes of medications. The results confirmed that undocumented migrants were less likely to be treated with intermediate- and long-acting insulins (PRR = 0.61, 95% CI: 0.49 to 0.76) and with metformin (PRR = 0.86, 95% CI: 0.77 to 0.96). In contrast, sulfonylureas were more frequently used among undocumented migrants than Italians (PRR = 1.74, 95% CI: 1.21 to 2.49).

### 3.2. Cardiovascular Drugs

These medications were more frequently prescribed to Italian diabetic patients. This was observed both in general and for all the different ATC subgroups; no patients in either group received C05 drugs (Table 3).

**Figure 2:**
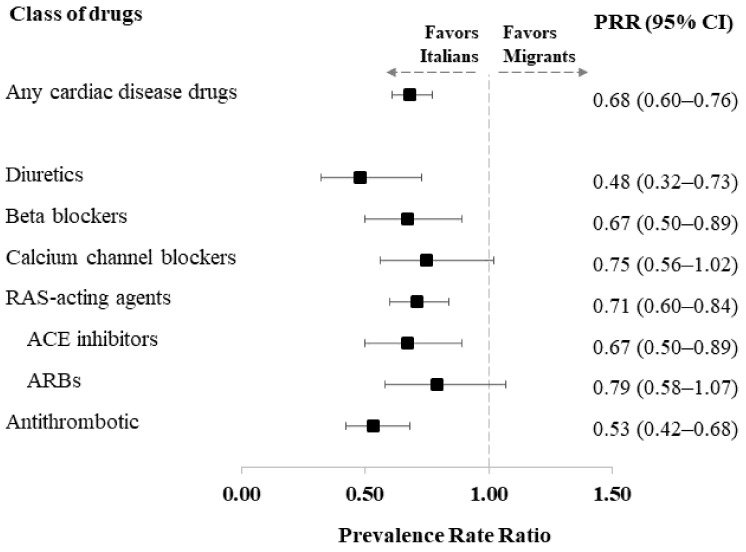
Prevalence rate ratio (PRR), along with 95% confidence intervals, estimated from a Poisson regression model for comparing the prevalence of use of selected cardiovascular therapy drugs between undocumented migrants and Italians, adjusted for patients’ characteristics (age class, sex, comorbidities, and Q-score).

Figure 2 shows the PRRs of the use of the different classes of CV drugs in migrants compared to Italians. 

This analysis confirmed that Italians received more CV medications. After correction for the aforementioned confounding factors, a lower use of all the classes of drugs considered was observed among undocumented migrants. The most marked differences were recorded for diuretics (PRR = 0.48, 95% CI: 0.32 to 0.73) and antithrombotics (PRR = 0.53, 95% CI: 0.42 to 0.68).

### 3.3. Sensitivity Analysis

The results obtained in the main analysis did not substantially change after excluding confounders with the higher rate of missing data from the Poisson model (data not shown). 

## 4. Discussion

In this study we have shown that the drug treatment of diabetes and its cardiovascular comorbidities was different between Italians and undocumented migrants. More medications were used to treat diabetes in Italian patients, with the exception of sulfonylureas and DPP-4 inhibitors, which were more frequently prescribed to migrants. This is quite puzzling in the light of international recommendations which are still placing metformin in first place when switching a patient without previous cardiovascular events from lifestyle interventions to pharmacological treatment [15,16,17]. The different use of antidiabetic medications in the two groups could be explained by the many different features between undocumented migrants and Italians. However, the differences in pharmacological treatment remained even after correcting for the main confounding factors. Moreover, it is strange that sulfonylureas were more frequently prescribed to undocumented migrants. These drugs are known to cause weight gain and have a significant risk of causing hypoglycemia [18]. This would not put them among the preferable options for patients, as undocumented migrants have many problems in accessing health services and in following healthy alimentary principles. We have no explanation for this observation. DPP-4 inhibitors carry a very low risk of hypoglycemia and also exert anti-atherosclerotic effects through many mechanisms [19], which makes them more suitable for these patients. The fact that they were less frequently prescribed to Italians could be counterbalanced by a more frequent use of SGLT-2 inhibitors, possibly because Italian patients had a greater prevalence of CV diseases and these medications are now recommended even as first-line therapy in this case [16,18]. Interestingly, sulfonylureas and DPP-4 inhibitors were the most prescribed second-line antidiabetic medications in some populations of the DISCOVER study [20]. GLP-1 analogues could be found only in the group of Italian patients. These molecules are positively associated with a reduction in major cardiovascular events in diabetics, renal protection, and weight loss [21]. Therefore, they appear to be indicated in different phenotypes of diabetes [22], which could be found both in Italians and undocumented migrants. It is tempting to consider that the reason for the difference in their use is that they are injective, and their storage and self-administration can be quite difficult for undocumented migrants, who live in precarious and overcrowded setting or are homeless. Should this be the reason, it does not yet explain the distribution of insulin therapy in the two groups.

The considerations made above are far from explaining the differences in antidiabetic therapy between our groups of Italians and undocumented migrants with diabetes. Though the latter might have a different prevalence of diabetic phenotypes [13], we have already demonstrated that pharmacologic treatment of diabetes does not appear to be based on disease phenotypes [23] and the present observations also do not point in the direction of phenotype-based therapeutic decisions. To an even lesser extent do they seem to be explained by efforts to take into account the potential pharmacogenetics aspects of diabetes [24]. More likely, within still suboptimal drug treatment of diabetes in general [25], they can be due first to differences in the preceding history of the disease in the two groups and second to different prescription habits and compliance to therapy in the two clinical settings. For the first point, it should be noted that, due to the design of our study, we have no data on the previous history of our patients, and this entails some limitations. For example, we do not know if the treatment at the moment of the observation was a first- or second- or even third-line treatment. Indeed, it is often necessary to change the type of antidiabetic medication, either due to the occurrence of side effects or due to the failure to obtain adequate glycemic control, which is a long-known problem [26]. For the second aspect, it could be that doctors working for OSF feel more uncomfortable when prescribing medications than their colleagues in a university hospital who deal with well-informed, easily contactable patients, who in the absence of the diabetologist can always rely on their family doctor. On the contrary, OSF patients are less informed, less easily contactable, and often do not even speak Italian, let alone having the possibility to contact a family doctor. These patients have to face many barriers in their everyday life, including difficulties in accessing medical assistance if not from charities. Moreover, being such an instable population, it is very hard to assess their level of compliance and adherence to treatment. These social problems could impact on their antidiabetic therapy in many ways, including therapeutic inertia [27]. This in turn could lead to less intensive treatment with less satisfactory control of the disease, as indicated by lower Q-scores and higher levels of glycated hemoglobin (HbA1c). Since HbA1c is a useful marker of diabetes progression and is associated with atherosclerotic complications, especially CV events, in which it is also suspected to have a causative role [28,29], this raises another issue with the population of this study. They more frequently had the typical complications of diabetes, such as renal involvement and diabetic skin ulcers, but less frequently had CV comorbidities, possibly also in relation to the fact that they had fewer cardiovascular risk factors and lower body mass indexes (BMIs). They also received fewer CV medications than Italian patients, including anti-hypertensive drugs. Undocumented migrants were younger, but this difference remained after correcting for confounding factors. This observation seems to favor the hypothesis of different diabetes phenotypes among undocumented migrants, which is also supported by other data such as a more frequent family history of diabetes and a younger age at diagnosis. The latter poses a question: is it possible that in certain ethnic groups, in spite of an earlier onset of diabetes, CV comorbidities take longer to develop, but they can eventually take place? 

Another cause of concern could be the fact that in some non-Western populations, diabetes has a very high prevalence. It peaks at 12.8% in persons aged 20–79 years in the Middle East and North Africa [30]. Some ethnic groups such as South Asians have an increased risk of developing type 2 diabetes and CV diseases at lower body mass indexes [31]. In the United States, diabetes is much more frequent in Black and Hispanic adults than in Whites of the same age [9]. In general, migrants to Europe have a high frequency of diabetes [32]. A greater prevalence of diabetes in migrant populations, beside other diabetes-related clinical problems, could also increase the impact of CV complications at a population level.

## 5. Conclusions

In this study, the pharmacological treatment of diabetes was different between patients of the host country and undocumented migrants. In general, the latter received fewer medications and had poorer glycemic control. We could not completely elucidate the reasons of these differences, but it is possible that socioeconomic and cultural factors have a role.

CV medications, too, were more frequently prescribed to Italian patients than undocumented migrants. This was in keeping with the reduced frequency of CV comorbidities in undocumented migrants, but it should be considered that CV complications could take longer to appear in certain ethnic groups. 

It is now known that many migrant populations have a very high prevalence of diabetes. This could mean that in the near future host countries could have to face CV and other complications of diabetes with greater frequency. To contend with this, some strategies could be readily implemented. For example, undocumented migrants have many opportunities to access the accident and emergency departments of Italian NHS hospitals; if a chronic condition is recognized, the patient could be referred to one of the NGOs operating in the area. Another opportunity would be to give undocumented migrants the possibility to take part in educational programs on chronic diseases held by scientific societies and patient associations. Much more could be accomplished and now, making more efforts to adequately cure these patients appears to be a rewarding investment for them and for public health.

## Figures and Tables

**Table 1:** Characteristics of 838 diabetic subjects included in the study.

Characteristics	Total Subjects	Undocumented Migrants	Italians	Missing Data	*p*-Value
	*n* = 838	*n* = 413	*n* = 425		
Age in years, median (IQR)	60.0 (15)	54 (15)	65 (14)		<0.0001
<50	160 (19.1)	138 (33.4)	22 (8.2)		<0.0001
50–59	245 (29.2)	155 (37.5)	90 (21.2)		
60–69	246 (29.1)	85 (20.6)	161 (37.9)		
≥70	187 (22.3)	35 (8.5)	152 (35.8)		
Age at diagnosis, median (IQR)	49 (15)	45 (15)	52 (14)	72	<0.0001
Female	334 (39.9)	171 (41.4)	163 (38.4)		0.3671
Nationality, *n* (%)					-
Italian	425 (51.2)	-	425 (100.0)		
Eastern Europe	95 (11.5)	95 (23.5)	-		
Mediterranean Africa	63 (8.6)	63 (15.6)	-		
Sub-Saharan Africa	37 (4.5)	37 (9.2)	-		
Central Asia	1 (0.1)	1 (0.3)	-		
South Asia	80 (9.6)	80 (19.8)	-		
East Asia	28 (3.4)	28 (6.9)	-		
Latin America	101 (12.2)	101 (24.9)	-		
BMI, mean (SD)	29.4 (6.4)	28.6 (6.9)	30.2 (5.9)	30	0.0003
Familiarity for diabetes	509 (65.3)	181 (43.8)	328 (77.2)	58	<0.0001
Cardiovascular disease risk factors	731 (88.0)	329 (79.7)	402 (94.6)	7	<0.0001
Risk behaviors	253 (30.2)	114 (27.6)	139 (32.7)	142	<0.0001
Hospitalization for diabetes	141 (17.0)	129 (31.2)	12 (2.8)	7	<0.0001
HbA1C, mean (SD)	7.8 (1.8)	8.6 (2.1)	7.2 (1.1)	66	<0.0001
Glycosuria	238 (31.8)	148 (35.8)	90 (21.2)	90	<0.0001
Ketonuria	18 (2.3)	14 (3.4)	4 (0.9)	96	0.0030
Cardiovascular disease	195 (23.3)	79 (19.1)	116 (27.3)	2	0.0058
Diabetic nephropathy	131 (16.3)	80 (19.4)	51 (12.4)	34	0.0025
Diabetic retinopathy	166 (21.2)	79 (19.1)	87 (20.5)	54	0.7353
Diabetic neuropathy	82 (11.6)	29 (7.0)	53 (17.2)	129	<0.0001
Ulcers	50 (6.1)	41 (9.9)	9 (2.1)	15	<0.0001
Q-score, mean (SD)	23.3	22.2 (9.3)	24.4 (8.6)		0.0005

**Table 2:** T2DM drugs used for undocumented migrants and Italians with diabetes.

Type of Drug	ATC Code	Total Subjects	Undocumented Migrants	Italians	*p*-Value
		*n* = 838	*n* = 413	*n* = 425	
Any oral diabetes medications		643 (76.9)	299 (72.6)	344 (81.1)	0.0033
Insulins and analogues for injection, fast-acting	A10AB	216 (25.8)	111 (26.9)	105 (24.8)	0.4850
Insulins and analogues for injection, intermediate- or long-acting combined with fast-acting	A10AE, A10AD	344 (41.1)	139 (33.7)	205 (48.4)	<0.0001
Metformin	A10BA02	581 (69.3)	263 (63.7)	318 (74.8)	0.0005
Sulfonylureas	A10BB, A10BC, A10BD01, A10BD02, A10BD04, A10BD06	143 (17.1)	85 (20.6)	58 (13.7)	0.0076
Glinides	A10BX02, A10BX03, A10BX08, A10BD14	13 (1.6)	2 (0.5)	11 (2.6)	0.0137
Alpha glucosidase inhibitors	A10BF	16 (1.9)	6 (1.5)	10 (2.4)	0.3411
Thiazolidinediones	A10BG	11 (1.3)	3 (0.7)	8 (1.9)	0.1416
Dipeptidyl peptidase 4 (DPP-4) inhibitors	A10BH, A10BD07, A10BD08; A10BD10, A10BD11, A10BD13, A10BD18	96 (11.5)	54 (13.1)	42 (9.9)	0.1468
Glucagon-like peptide-1 (GLP-1) analogues	A10BJ, A10BX04, A10BX07, A10BX10, A10BX13, A10BX14	59 (7.0)	0 (0.0)	59 (13.9)	<0.0001
Sodium–glucose co-transporter 2 (SGLT2) inhibitors	A10BK, A10BX09, A10BX11, A10BX12, A10BD15, A10BD16, A10BD20	52 (6.2)	7 (1.7)	45 (10.6)	<0.0001
Other blood glucose-lowering drugs, excl. insulins	A10BX	31 (3.7)	16 (3.9)	15 (3.5)	0.7916

**Table 3:** CV drugs used for undocumented migrants and Italians with T2DM.

Disease	ATC Code	Total Subjects	Undocumented Migrants	Italians	*p*-Value
		*n* = 838	*n* = 413	*n* = 425	
Any heart and vessels disease drugs	C01, C02, C03, C05, C07, C08, C09, B01	564 (67.3)	210 (50.9)	354 (83.3)	<0.0001
Cardiac therapy	C01	53 (6.3)	17 (4.1)	36 (8.5)	0.0096
Antihypertensives	C02	35 (4.2)	3 (0.8)	32 (7.5)	<0.0001
Diuretics	C03	136 (16.2)	30 (7.3)	106 (24.9)	<0.0001
Vasoprotectives	C05	0	-	-	-
Beta blockers	C07	205 (24.5)	69 (16.7)	136 (32.0)	<0.0001
Calcium channel blockers	C08	198 (23.6)	80 (19.4)	118 (27.8)	0.0042
Renin–angiotensin system (RAS)-acting agents	C09	440 (52.5)	166 (40.2)	274 (64.5)	<0.0001
ACE inhibitors	C09A, C09B	234 (27.9)	85 (20.6)	149 (35.1)	<0.0001
ARBs	C09C, C09D	212 (25.3)	84 (20.3)	128 (30.1)	0.0011
Antithrombotics	B01	271 (32.3)	73 (17.7)	198 (46.6)	<0.0001

## Data Availability

The data used to support the findings of this study are available from the corresponding author on reasonable request.
